# Microbiome and Metabolome Variation as Indicator of Social Stress in Female Prairie Voles

**DOI:** 10.3390/ijms24021677

**Published:** 2023-01-14

**Authors:** Daniel A. Nuccio, Marigny C. Normann, Haiming Zhou, Angela J. Grippo, Pallavi Singh

**Affiliations:** 1 Department of Biological Sciences, Northern Illinois University, Dekalb, IL 60115, USA; 2 Department of Psychology, Northern Illinois University, Dekalb, IL 60115, USA; 3 Department of Statistics and Actuarial Sciences, Northern Illinois University, Dekalb, IL 60115, USA

**Keywords:** social isolation, prairie voles, gut microbiome, gut–brain axis, colitis, type two diabetes

## Abstract

Social isolation is detrimental to the health of social mammals inducing neurochemical and hormonal changes related to depression and anxiety, as well as impairments of cardiovascular and immune functioning. Likewise, perceptions of loneliness are increasingly recognized as detrimental to human psychological well-being, cognitive functioning, and physical health. Few studies, however, have examined the impact of social isolation on the intestinal microbiome and metabolome. To better understand the impact of social isolation on these systems, intestinal microbiota, and the systemic impact via the gut–brain axis, we employed prairie voles. Physiological stress on female prairie voles (n = 22) either with a same-sex sibling (n = 11) or in isolation (n = 11) for four weeks demonstrated behavioral indicators of increased anxiety and depression in isolated voles (*p* ≤ 0.01). Bacterial DNA from fecal and colon samples, collected at five time points (T_0–4_), were sequenced for all nine hypervariable regions of the 16S rRNA gene. Microbiome analyses revealed several differences in gut communities of paired and isolated voles with greater differences at T_4_. Notably, several taxa associated with host health including *Anaerostipes* and *Lactobacillaceae* were more prevalent in paired voles, whereas several taxa associated with known pathogens (e.g., *Staphylococcaceae* and *Enterococcus*) or disease were elevated in isolated animals. Similarly, metabolome analyses suggested isolated voles, when compared to paired animals, exhibited differences in metabolites associated with diabetes and colitis. These findings further contribute to our understanding of the harmful effects of social isolation, which cause perturbations in the gut microbiome and serum metabolites.

## 1. Introduction

In recent decades social isolation and perceptions of loneliness have been increasingly recognized as detrimental to the psychological well-being [1,2], cognitive functioning [2,3], and physical health of humans, as well as meaningful risk factors for premature death [4]. The psychological consequences of loneliness include increased depression, anxiety, aggression, impulsivity, and substance abuse [1]. Physical and mental stress due to loneliness has been associated with adverse health outcomes including obesity and poor cardiovascular health [1,2]. Furthermore, chronic stress can be detrimental to host health and be associated with poorer mental health and cognition, and an increased risk for pathology and disease [2,5,6]. For example, socially isolated naked mole-rats have sustained increases in fecal cortisol levels [7].

Individually housed mice predisposed to obesity and diabetes experience an accelerated onset of these conditions as well as relevant metabolic changes [8]. The post-weaning social isolation of rats leads to increases in behavioral measures of anxiety, decreases in associative learning and memory, reduced neurogenesis in the dentate gyrus of the hippocampus, and alterations in the gut microbiota [9].

One of the preferred model organisms for research on social isolation is the prairie vole (*Microtus ochrogaster*) on account of this species’ engagement in socially monogamous pair-bonds and biparental care, as well as their propensity to live in extended family groups [10,11,12]. Physiologically, isolated prairie voles exhibit changes in immune, cardiovascular, and neuroendocrine functioning [13,14,15]. Furthermore, isolated female prairie voles have been shown to have an increased heart rate, as well as decreased heart rate variability, potentially due to increased sympathetic nervous system (SNS) activity and decreased vagal control of the heart through the parasympathetic nervous system (PNS), both indicative of abnormal autonomic nervous system (ANS) functioning [16,17].

Several hormones have also been implicated as potential mechanisms for behavioral and cardiovascular changes. These include oxytocin (OT), vasopressin (AVP), corticotropin-releasing hormone (CRH), adreno-corticotrophin (ACTH), and corticosterone [13,15,18,19,20,21], all of which have been implicated in stress responses [5,6]. Isolated female prairie voles have increased levels of plasma OT [19,20], AVP [19], and CRH [19]. Although similar results are not always found in isolated male prairie voles, as female prairie voles are believed to be more susceptible to social stressors than males [20,22], males have exhibited increases in plasma OT and OT, AVP, and CRH immunoreactive cells in the paraventricular nucleus (PVN), an important control center of the brain [13,15,20].

Despite all the research conducted on the behavioral and physiological consequences of social isolation in prairie voles, however, few studies have examined the impact of social isolation on the prairie vole microbiome, gut–brain axis, and metabolome. To date, there are only a limited number of studies exploring the gut microbiome of prairie voles, and even fewer examining whether it is impacted by the stress of social isolation [18,23,24,25]. To our knowledge, the only previous study to examine the impact of social isolation on the gut microbiota of these animals is a paper from 2020 in which differences in microbial fecal communities of paired and isolated voles were reported following a six-week period [18].

To expand on these findings and better understand the effects of social isolation on intestinal microbial community and its role in stress induced state on host health we utilized female prairie voles on account of their previously noted increased sensitivity to social stressors compared to males [20,22]. Following four weeks of isolation, we tested animals for behavioral indicators of depression and anxiety, changes in gut microbiota, and differences in metabolites based on housing conditions. Collectively, this was done both for exploratory purposes and to search for possible links between behavior, gut microbiome composition, and fecal and serum metabolite concentrations following social isolation or paired housing. We hypothesized that the intestinal microbiome of paired voles would have a higher abundance of beneficial microbes, and that their metabolome would contain a greater level of metabolites associated with better health outcomes. Our findings support these hypotheses as isolated animals engaged in behaviors associated with anxiety and depression more than paired animals, and exhibited a greater abundance of bacterial taxa associated with poorer health and disease in their intestinal microbiota. Furthermore, although not significant, the metabolomes of isolated animals, when compared to those of paired animals, tended to show differences suggestive of poorer health.

## 2. Results

### 2.1. Weight and Anatomical Measures

No significant differences between paired and isolated voles were found for animal weight at the start or end of the experiment, nor was there a significant change in body weight between groups between these time points. Similarly, no significant differences between groups were found for adrenal:body weight ratio or heart:body weight ratio.

### 2.2. Behavioral Tests

**Figure 1:**
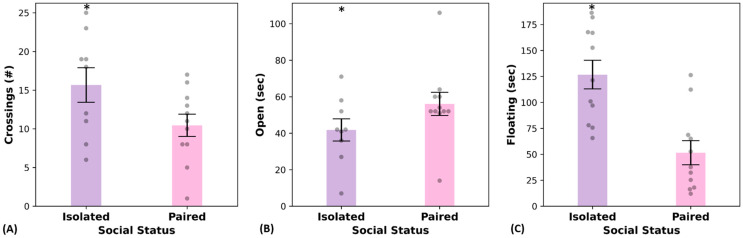
When placed in the elevated plus maze, socially isolated prairie voles exhibited behaviors associated with hyperactivity, anxiety, and depression. (**A**) Isolated voles exhibited a greater number of center crosses (N = 9, M = 15.667, SEM 2.224) than paired voles (N = 11, M = 10.45, SEM = 1.436). (**B**) Isolated voles spent less time in the open arms of the EPM (N = 9, M = 41.778, SEM = 6.105) than paired voles (N = 11, M = 56.091, SEM = 6.362). (**C**) When placed in the FST, isolated voles exhibited increased immobility (N = 11, M = 126.8, SEM = 13.8) compared to paired voles (N = 11, M = 51.5, SEM, 11.7). * denotes statistically significant differences (*p* < 0.05).

Isolated voles (n = 9), when compared to paired voles (n = 11), exhibited significantly higher number of center crosses in the EPM (*t* = −2.038, *p* = 0.028; Figure 1A) and decreased time in the open arms of the EPM (*t* = 1.772, *p* = 0.046; Figure 1B). They also displayed increased immobility in the FST (*t* = 4.17, *p* < 0.001; Figure 1C). Two animals from the isolated condition were excluded from EPM analyses because they fell off the apparatus.

### 2.3. Microbiome Analyses

Firmicutes (55.36%) and Bacteroidetes (39.44%) were the dominant microbial phyla in all fecal samples, irrespective of the treatment group, followed by *Actinobacteria* (1.82%), Proteobacteria (1.24%), and Candidatus Saccharibacteria (1.1%; Supplemental Figure S1). The most abundant families of Firmicutes in fecal samples were *Ruminococcaceae* (20.92%), *Lachnospiraceae* (11.02%), and *Erysipelotrichaceae* (24.44%). *Muribaculaceae* (31.07%) was the most prominent representative of the Bacteroidetes phylum (Supplemental Figure S2). The most prominent genera present were *Ileibacterium* (20.6%) and *Ruminococcus* (12.68%) from the Firmicutes phylum, as well as *Duncaniella* (26.62%) from the Bacteroidetes phylum.

When fecal community data from all time points were analyzed together, no differences were initially found between the communities of isolated and paired voles. However, LEfSe analyses found a greater proportional abundance of *Anaerostipes* in paired voles (LDA = 3.866, *p* = 0.027; Supplemental Figure S3) and greater proportional abundances of nine other taxa in isolated voles. Most notably, these include the family *Staphylococcaceae* (LDA = 2.178, *p* = 0.044) and the genera *Metaprevotella* (LDA = 2.028, *p* = 0.033) and *Enterococcus* (LDA = 2.2941, *p* = 0.039; Supplemental Figure S3). No significant differences were found for measures of alpha and beta diversity between isolated and paired animals.

### 2.4. Fecal Time Point Analysis

Microbial community members were found to differ between five (T_0_–T_4_) time points with significant differences in four families and eight genera (Table 1). Most notably these included Bdellovibrionaceae (*p* = 0.002) *Vampirovibrio (p* = 0.002), and *Anaerobutyricum* (*p* < 0.001).

**Figure 2:**
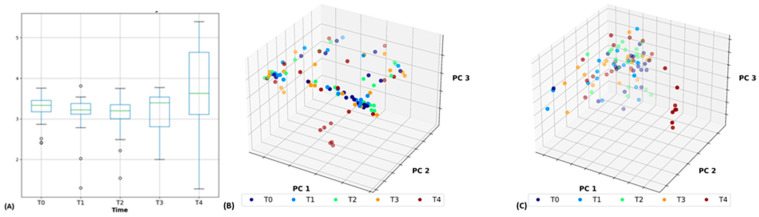
(**A**) Shannon diversity exhibited a trend toward significance with greater diversity driven by greater diversity being present at T4 than at T2 (*p* = 0.066). (**B**) Bray Curtis dissimilarity (*p* = 0.018), and (**C**) Jaccard distance (*p* = 0.001) demonstrate distinct groupings at T4.

Diversity analyses for time point data revealed a distinct grouping of taxa at T_4_ using Shannon diversity (*p* = 0.066; Figure 2A), Bray Curtis dissimilarity (*p* = 0.018; Figure 2B), and Jaccard distance (*p* = 0.001; Figure 2C) measures. Subsequent analyses of Shannon diversity using a Conover test demonstrated a trend toward higher diversity at T_4_ compared to T_2_ (*p* = 0.068). Further examination of the taxa identified as having significant differences between time points by Friedman tests revealed significant or close to significant differences in their respective proportional abundances at T_4_ and the remaining four time points.

**Figure 3:**
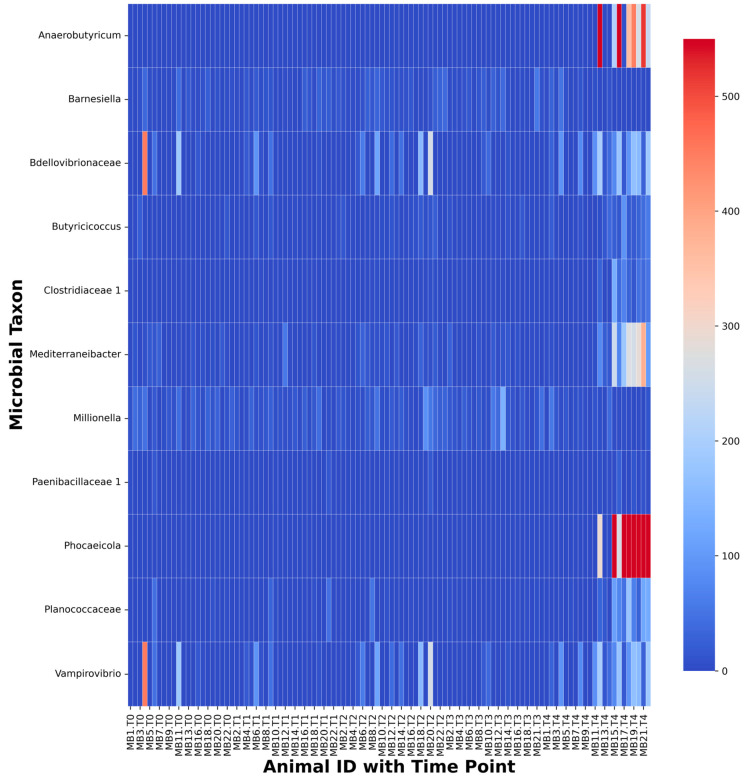
Heatmap depicting taxa that exhibited significant changes in abundance between five time points for all animals. Cells in red indicate higher abundance, whereas blue indicates lower abundance. The scale on the right indicates the heatmap scale. For clarity, *x*-axis labels show information for every other fecal sample included in the analysis. *Ruminococcus* were not included here, because the higher general abundance of this taxon would offset the scale of the heatmap.

These differences were driven by the families *Clostridiaceae* 1, *Paenibacillaceae* 1, *Planococcaceae*, and *Bdellovibrionaceae* and the genera *Anaerobutyricum*, *Phocaeicola*, and *Vampirovibrio,* most of which showed a greater proportional abundance at T_4_ than at previous time points (Figure 3). *Clostridiaceae* 1 had a greater proportional abundance at T_4_ than at T_0_ (*p* = 0.009), T_1_ (*p* = 0.034), T_2_ (*p* = 0.009), or T_3_ (*p* = 0.003). *Paenibacillaceae* 1 demonstrated a trend towards greater proportional abundance at T_4_ than T_0_ (*p* = 0.056) and a greater proportional abundance at T_4_ than T_1_ (*p* < 0.001), T_2_ (*p* = 0.004), or T_3_ (*p* < 0.001). *Planococcaceae* demonstrated a trend towards greater proportional abundance at T_4_ than T_0_ (*p* = 0.1) and a greater proportional abundance at T_4_ than T_1_ (*p* = 0.007), T_2_ (*p* = 0.007), or T_3_ (*p* = 0.017). *Bdellovibrionaceae* demonstrated a trend toward greater proportional abundance at T_4_ than T_0_ (*p* = 0.057) and a greater proportional abundance at T_4_ than T_1_ (*p* = 0.032), T_2_ (*p* = 0.032), or T_3_ (*p* < 0.001). *Anaerobutyricum* had a greater proportional abundance at T_4_ than at T_0_ (*p* < 0.001), T_1_ (*p* < 0.001), T_2_ (*p* < 0.001), or T_3_ (*p* < 0.001). *Phocaeicola* had a greater proportional abundance at T_4_ than at T_0_ (*p* = 0.002), T_1_ (*p* = 0.005), T_2_ (*p* = 0.007), or T_3_ (*p* < 0.001). *Vampirovibrio* showed a trend toward greater proportional abundance at T_4_ than T_0_ (*p* = 0.058) and a greater proportional abundance at T_4_ than T_1_ (*p* = 0.035), T_2_ (*p* = 0.035), or T_3_ (*p* < 0.001).

### 2.5. Stress-Associated Fecal Community Changes with Time

**Figure 4:**
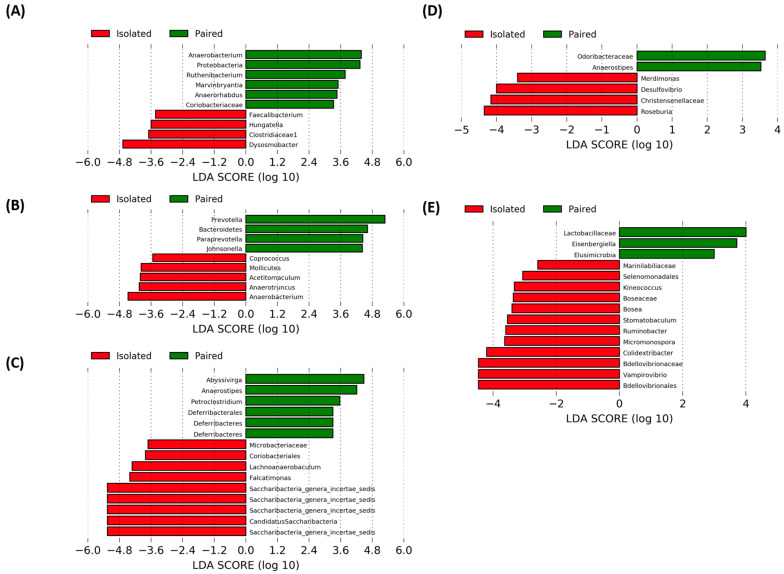
LEfSe analyses revealed differences in the proportional abundance of numerous taxa when the fecal communities of paired and isolated voles were compared at T_0_–T_4_. (**A**) At T_0_ notable differences include a greater proportional abundance of the phylum Proteobacteria at in paired voles (N = 11) compared to isolated voles (N = 10). (**B**) At T_1_ notable differences include a greater proportional abundance of Bacteroidetes, *Prevotella*, and *Paraprevotella* in paired voles (N = 11) compared to isolated voles (N = 11). (**C**) At T_2_ notable differences include a greater proportional abundance of *Anaerostipes* in paired voles (N = 11) compared to isolated voles (N = 11). (**D**) At T_3_ notable differences include a greater proportional abundance of *Anaerostipes* in paired voles (N = 11) compared to isolated voles (n = 10). (**E**) At T_4_, notable differences include greater proportional abundances of *Lactobacillaceae* in paired voles (N = 11) and *Bdellovibrionales, Bdellovibrionaceae,* and *Vampirovibrio* in isolated voles (N = 11).

Upon comparing fecal communities of isolated and paired voles at each time point using Mann-Whitney U tests, no significant differences in proportional abundances were shown between isolated and paired animals were found. However, a trend towards an increase in proportional abundance of *Prevotellaceae* in paired voles at T_1_ and the genus *Anaerostipes* at T_2_ were observed (Supplemental Table S1). Similarly, differences were found between the fecal communities of isolated and paired voles using LEfSe, in 10, 9, 15, 6, and 15 taxa at T_0_, T_1_, T_2_, T_3_, and T_4_, respectively (Figure 4A–E). Notably these include a greater proportional abundance of the phylum Proteobacteria at T_0_ in paired voles (LDA = 4.333, *p* = 0.041), the phylum Bacteroidetes (LDA = 4.62, *p* = 0.045) and member taxa *Prevotella* (LDA = 5.283, *p* = 0.045) and *Paraprevotella* (LDA = 4.441, *p* = 0.028) in paired voles at T_1_, a greater proportional abundance of *Anaerostipes* in paired voles at T_2_ (LDA = 4.212, *p* = 0.027) and T_3_ (LDA = 3.526, *p* = 0.04), and, at T_4_, greater proportional abundances of the family *Lactobacillaceae* (LDA = 4.006, *p* = 0.014) in paired voles and the order *Bdellovibrionales *(LDA = 4.463, *p* = 0.044), family *Bdellovibrionaceae* (LDA = 4.463, *p* = 0.044), and genus *Vampirovibrio* (LDA = 4.463, *p* = 0.044) in isolated voles.

### 2.6. Colon Microbial Community Analysis

We also compared individual animals and followed changes through the time points in each of the groups. Taxa less than 0.005% of any fecal sample community were filtered and then categorized taxa by their OTU abundance into three ranges (≤1000, 1001–10,000, and ≥10,001) for representation on heatmaps. At the genus level, taxa with OTU abundance in the 1001–10,000 range were highly variable by the host in both the groups across time points (Supplemental Figure S4 for isolated voles and 5 for paired voles). Most of the taxa identified in Section 2.4 above are noted within individual animals on the heat maps as well. Most variations started emerging at T3 for some but at T4 for all animals. At the genus level *Muribaculum*, *Dubosiella*, and *Intestimonas* increased in abundance at T4 in paired voles whereas isolated animals saw a decrease. Although *Lactobacillus* trends towards an increase in the isolated voles, the abundance of this taxa is higher in paired animals and fell in the final abundance range ≥10,001. Hence, for paired voles, *Lactobacillus* was not included in the figure displaying taxa with a maximum OTU count of 1001–10,000 reads per taxa (Supplemental Figure S5).

Firmicutes (54.04%) and Bacteroidetes (33.9%) were the dominant phyla found in colon communities. These were followed by *Actinobacteria* (7.5%), *Verrucomicrobia* (1.71%), and Proteobacteria (1.06%; Supplemental Figure S6). The most abundant families from the Firmicutes phylum were *Ruminococcaceae* (29.34%), *Lachnospiraceae* (14.95%), and *Erysipelotrichaceae* (7.5%) (Supplemental Figure S7). The most abundant families from the Bacteroidetes phylum were *Muribaculaceae* (12.19%) and *Bacteroidaceae* (6.24%; Supplemental Figure S5*)*. The dominant genera in the colon were *Olsenella* (9.69%) from the *Actinomycetes* phylum and *Duncaniella* (10.32%) from the *Muribaculaceae* family. Other prominent genera included the *Ileibacterium* (5.29%) from the Firmicutes phylum and *Alistipes* (6.37%), *Phoecaecola* (5.72%), and *Mediterranea* (5.28%) from the Bacteroidetes phylum. No significant differences in taxa were observed between the colon and T_4_ fecal communities. However, *Prevotellaceae, Streptococcaceae*, *Clostridiales Incertae Sedis XIII*, *Enterobacteriaceae*, *Akkermansiaceae*, *Bifidobacteriaceae*, and *Subdivision 5 Incertae Sedis* had a greater proportional abundance in colon samples. At the genus level, 98 community members differed in abundance with most of these showing a greater proportional abundance in colon samples. LEfSe analyses, however, showed 168 differences in proportional abundances of taxa in the colon and T_4_ fecal communities with 132 of those taxa having a greater proportional abundance in colon samples (Supplemental Figure S8).

**Figure 5:**
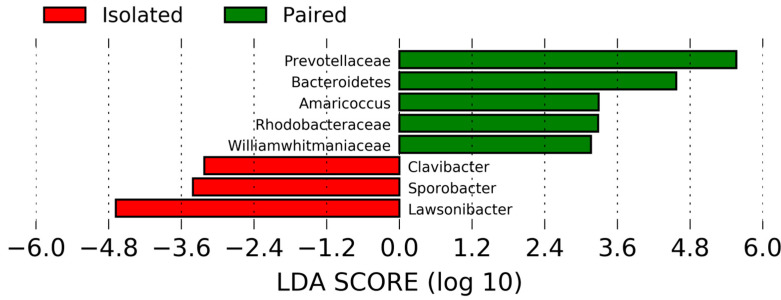
LEfSe analyses demonstrated differential abundance of microbial community members in colons of paired (N = 11) and isolated voles (N = 11). Bacteroidetes, *Prevotellaceae*, *Rhodobacteraceae*, *Williamwhitmaniaceae*, and *Amaricoccus* exhibited a greater abundance in paired voles and *Clavibacter*, *Sporobacter*, and *Lawsonibacter* in isolated voles.

Likewise, no significant differences in colon communities of isolated and paired voles were observed (Supplemental Table S1). Although, isolated voles exhibited a greater proportional abundance of the Firmicutes phylum and the genus *Lawsonibacter*. In addition, LEfSe revealed a greater abundance of Bacteroidetes (LDA = 4.57, *p* = 0.003) and *Prevotellaceae* (LDA = 5.5634, *p* = 0.02) in paired voles and *Lawsonibacter* (LDA = 4.682, *p* = 0.045) in isolated voles (Figure 5). No significant differences were found for the alpha or beta diversity between the colon communities of isolated and paired voles.

### 2.7. Metabolite Profiling

Overall, 231 fecal metabolites and 174 serum metabolites were detected. Fecal metabolites included 20 LCFAs and seven SCFAs; serum metabolites included 13 LCFAs and six SCFAs. No significant differences were found in the fecal or serum metabolites of isolated and paired animals at any time point. Likewise, no significant changes in metabolites were found when T_0_ values were subtracted from T_4_ values. However, we note several non-significant trends were observed. At T_4_ tetradecanoic acid, succinic acid, glucose, arabinose, galactose, hippuric acid, N-acetyl glucosamine, oxalic acid, and o-phosphoethanolamine were higher in fecal collections from isolated voles (n = 9), while hippuric acid was higher in paired animals (n = 11; Supplemental Table S2). Upon subtracting baseline T_0_ values from corresponding T_4_ values for fecal metabolites and comparing changes in the metabolites of paired and isolated animals, no differences were found. When serum samples were examined, although not statistically significant, hippuric acid and lactic acid were found to be higher in paired voles (n = 11), whereas sorbitol and glyoxylic acid were higher in isolated animals (n = 11; Supplemental Table S3).

### 2.8. Associations of Anxiety and Depressive-like Behaviors with Multi-Omics Data

Correlations between behavioral measures at T_4_ were observed for isolated and paired voles_._ In paired voles, positive correlations were found between time spent in the open arms of the EPM and serum concentrations of both tetradecanoic acid (r = 0.793, *p* = 0.004) and butanoic acid (r = 0.777, *p* = 0.005), as well as between immobility in the FST and fecal tetradecanoic acid (r = 0.851, *p* = 0.001). Additionally, 23 colon taxa and 46 fecal taxa were either positively or negatively correlated with behavioral measures (Supplemental Table S4). Consistently, in samples from isolated animals, negative correlations were found between time spent in the open arms of the EPM and *Jonesiaceae* (*r* = −0.750, *p* = 0.019) and *Sanguibacter* (*r* = −0.750, *p* = 0.019), both members of the phylum Actinobacteria; although interestingly, in paired animals, positive correlations were found between time spent in the open arms of the EPM and members of these same taxa, *Jonesiaceae r* = 0.784, *p* = 0.004) and *Sanguibacter (r* = 0.784, *p* = 0.004).

## 3. Discussion

Social isolation is a serious threat to the normal physiological functioning and the psychological well-being of social animals. The gut microbiome and the nervous system engage in bidirectional communication facilitated by the vagus nerve [26,27], sensory nerves [27], immune cell secretions [28], hormone-like molecules produced by microbes [29], and gastrointestinal secretions [27]. Therefore, in this study, we examined the effects of social isolation on the prairie vole gut microbiome and determined the possible role of fecal and serum metabolites in mediating changes in host health through the gut–brain axis.

### 3.1. Social Isolation-Induced Behaviors Associated with Anxiety and Depression

Behaviorally, isolated prairie voles have been shown to exhibit increased immobility in the FST [30], decreased time in the open arms of the EPM [15,21,30], and an increased likelihood of attacking other voles when briefly placed with one in an enclosure [15,31]. Together, these behaviors indicate greater signs of depression, anxiety, and aggression in isolated prairie voles. Utilizing prairie voles, long recognized for their highly social lifestyle, we implemented a social isolation procedure that induced behavioral indicators of depression and anxiety. These indicators have previously been reported to be manifested in conjunction with the abnormal functioning of several physiological systems including the cardiovascular and immune systems. Our results following this four-week study, were consistent with those of these past studies based on the FST and EPM behavioral tests, suggesting isolated animals in our study, following a four-week isolation period, were exhibiting behavioral changes associated with depression and anxiety through our implementation of a social isolation paradigm.

### 3.2. Prairie Vole Fecal Microbiome Changes with Time

To date, there are only four studies directly examining the prairie vole gut microbiome or its members [18,23,24,25]. In our study, we analyzed intestinal microbial composition based on all nine hypervariable regions of the 16S rRNA gene, which had not been previously done using prairie voles. To our knowledge, our study is also the first study to analyze differences with frequent sample collection: once per week for four weeks.

Using pooled data from all four time points, we found Firmicutes (55.36%) and Bacteroidetes (39.44%) to be the dominant phyla in prairie vole fecal communities, followed by *Actinobacteria* (1.82%), Proteobacteria (1.24%), and Candidatus Saccharibacteria (1.1%). At the family level, we found *Muribaculaceae* (31.07%), *Erysipelotrichaceae* (24.44%), and *Ruminococcaceae* (20.92%) to be the dominant taxa, followed by *Lachnospiraceae* (11.02%), *Prevotellaceae* (2.14%), *Lactobacillaceae* (1.44%), *Saccharibacteria genera incertae sedis* (1.28%), and *Rickenellaceae* (1.24%). These results are broadly similar to those of previous studies that sought to characterize the prairie vole gut microbiome using fecal samples [18,23].

Bray Curtis dissimilarity and Jaccard distance measures revealed that prairie vole fecal communities at T4 clustered separately from those of T_0_–T_3_ (Figure 2B,C). Friedman tests and subsequent Conover tests suggested that these differences may have been driven, in part, by increases in the families *Clostridiaceae* 1, *Paenibacillaceae* 1, *Planococcaceae*, and *Bdellovibrionaceae*, as well as the genera *Anaerobutyricum*, *Phocaeicola*, and *Vampirovibrio* (Figure 3). *Anaerobutyricum* is a common member of the mammalian gut microbiome capable of producing SCFAs [32]. These metabolites can be used as an energy source by colonocytes [33] and are hypothesized to play a role in type two diabetes risk [34], inhibit the development of colorectal cancer [35], and prevent an array of autoimmune and gastrointestinal disorders [36]. More specifically, *Anaerobutyricum soehngenii* can convert sucrose or acetate and sorbitol to butyrate and formate, as well as acetate and lactate to butyrate [32]; *Anaerobutyricum hallii* is likely also capable of converting acetate and lactate to butyrate [32].

*Vampirovibrio* also contributed to the clustering of T_4_ fecal communities from those of other time points, however, its role in the mammalian gut microbiome is less clear, as is its classification. The genus *Vampirovibrio* contains the species *Vampirovibrio chlorellavorus* which engages in a predatory lifestyle [37] and is still assigned by some classification tools (e.g., the RDP Classifier version 2.13) to the order *Bdellovibrionales* and family *Bdellovibrionaceae*, which contain other predatory species of bacteria that have been suggested to have potential as tools in controlling drug-resistant Gram-negative pathogens [38,39]. However, in 2015 researchers argued that *Vampirovibrio* belongs to its own family and order within the *Melainabacteria* taxon [37], which was initially suggested as a sister phylum of *Cyanobacteria* [40] but has more recently been treated as a class of *Cyanobacteria* [37]. Members of the *Melainabacteria* taxon are believed to be non-photosynthetic obligate anaerobic fermenters capable of utilizing a wide variety of carbon sources including polysaccharides, oligosaccharides, simple sugars, amino acids, fatty acids, and organic acids to produce lactate, formate, ethanol, and possibly butyrate [40]. Investigations of archival databases of microbiome data have suggested *Melainabacteria* may be somewhat common members of the gut microbiomes of animals, including humans, with diets containing large amounts of plant-based fiber [40]. Interestingly, members of the *Melainabacteria* order *Gastranerophiliales* have been found in the fecal communities of prairie voles by both of the previous studies to characterize the prairie vole gut microbiome [18,23], suggesting they may be common members of the prairie vole fecal microbiome. Previous research has suggested that *Melainabacteria* members may benefit their hosts through the biosynthesis of several vitamins including riboflavin, nicotinamide, biotin, and dihydrofolate [40]. Conversely, it is not clear that *Vampirovibrio* shares the same abilities and potential benefits of other *Melainabacteria* members. Furthermore, evidence is emerging that increases in the abundance of *Cyanobacteria* in gut communities is correlated with gastrointestinal and neurodegenerative disease [41]. For example, increased abundances of *Vampirovibrio*, specifically, have been found in colorectal fecal communities in a rat model of irritable bowel syndrome established by subjecting rats to multiple forms of stress, including early maternal separation [42]. Additionally, it is worth noting that, the one other study to compare the gut communities of paired and isolated prairie voles, found increases in the abundance of a *Gastranaerophilales* gut metagenome species in isolated animals [18].

### 3.3. Isolation-Induced Changes in Gut Taxa

When differences in the gut microbiota of paired and isolated voles were examined, consistent with our hypotheses, analyses revealed those of paired voles to contain a greater abundance of taxa that may be favorable to host health, whereas those of isolated voles were revealed to contain more taxa associated with pathogens and disease, which may be early markers of disease conditions. This may be another contributing factor to the divergence of microbial communities of the animals at T4 which clustered separately from those of T_0_–T_3_ (Figure 2B,C). In paired voles, we recorded a greater proportional abundance of *Anaerostipes*, most prominently at T_2_ and T_3_, and a greater proportional abundance of *Lactobacillaceae* at T_4_. In isolated voles, some of the most interesting community features observed included a greater proportional abundance of *Enterococcus*, *Staphylococcaceae*, and *Metaprevotella* in pooled data from all five time points, and a greater proportional abundance of *Bdellovibrionales*, *Bdellovibrionaceae*, and *Vampirovibrio* at T_4_. Broadly, our results showed some similarities to a previous study which was based on sequencing of the V3–V4 regions [18], in which the authors reported that isolated animals exhibited decreases in three taxa with growth promoting effects: *Anaeroplasma, Ruminococcaceae UCG-014*, and *Butyrivibrio.*

Of the notable taxa that differed between paired and isolated voles, *Anaerostipes* and *Lactobacillaceae*, which had a greater abundance in the fecal communities of paired voles, have previously been associated with putative benefits to host health either through the production of short chain fatty acids (SCFAs) [43,44,45] or regulation of pathogen growth and colonization [23,46]. Conversely, several taxa, such as *Enterococcus*, *Metaprevotella*, and *Staphylococcaceae* that had a greater abundance in the fecal communities of isolated voles have previously been associated with disease [35,42,47,48,49].

*Anaerostipes caccae*, when co-cultured with *Bacteroides thetaiotamicron*, produces butyrate from monosaccharides, lactate, and acetate produced from *Bacteroides thetaiotamicron* [44]. *Anaerostipes rhamnosivorans* produces butyrate when grown alone and propionate when co-cultured with *Bifidobacterium longum* subsp. *infantis* [43]. Furthermore, when co-administered with myo-inositol for six weeks to mice fed a Western diet, fasting glucose levels in these mice were reduced, suggesting *A. rhamnosivorans* may play a role in preventing the onset of diabetes or a future treatment for the condition [43]. This potential role for *Anaerostipes* is strengthened by previous research that demonstrated African patients with type two diabetes had lower abundances of *Anaerostipes* in their gut microbiomes than healthy controls [50]. Members of the family *Lactobacillaceae* have long been recognized for their probiotic potential, showing the ability to adhere to and aggregate on epithelial cell lines and inhibit the growth of such pathogens as *Escherichia coli, Salmonella typhimurium,* and *Enterococcus faecalis* [46]. Additionally, previous research specifically examining the probiotic potential of 30 *Lactobacilli* isolated from the prairie vole intestine, found five strains with antimicrobial properties against *E. coli*, *Pseudomonas aeruginosa*, *Staphylococcus aureus*, and *Candida albicans*, as well as the ability to adhere to intestinal epithelial cells and survive in the presence of bile and in low pH environments [23]. Additionally, decreased abundance of *Lactobacillaceae* has been associated with end-stage renal disease [34].

Conversely, members of the family *Staphylococcaceae* and genus *Enterococcus* have long been associated with host disease. An elevated abundance of *Staphylococcaceae* has been associated with the development of colorectal cancer [35]. The genus *Enterococcus* includes members linked to colorectal cancer, inflammatory disease, and a variety of gastrointestinal disorders [47]. An increased abundance in the family *Enterococcaceae* has also been associated with the increased severity of certain symptoms in Parkinson’s patients [49]. Although little is known about the recently discovered genus *Metaprevotella*, the first studied *Metaprevotella* strain was initially isolated from the ileum of a Crohn’s patient [48]. The differences we uncovered in the fecal communities of paired and isolated voles demonstrate a trend in which at least two taxa with a greater proportional abundance in the fecal communities of paired voles are thought to provide a benefit to host health, whereas several with a greater proportional abundance in the fecal communities of isolated voles have members that are either considered pathogens or associated with disease, most notably, type two diabetes and various gastrointestinal disorders.

### 3.4. Prairie Vole Colon Communities Differ from Fecal Communities

Upon investigating prairie vole colon communities, we found general similarities in the abundances of the most prominent phyla compared to the T_4_ fecal communities of these animals, but noted numerous differences, especially at the levels of family and genus. Furthermore, when comparing colon communities of paired and isolated voles, we found differences distinct from those found when the fecal communities of paired and isolated animals were compared. Notably, among the differences uncovered in colon communities were an increased proportional abundance of the phylum Bacteroidetes and the family *Prevotellaceae* in paired voles and the genus *Lawsonibacter* in isolated voles. However, the manner in which these differences may have had an impact on the health of our animals remains unclear. Members of the *Prevotellaceae* genus *Prevotella*, although associated with various inflammatory conditions, are also a common part of the human microbiome, often showing an increased abundance in the gut microbiomes of vegetarians and non-Westerners with plant-rich diets [51]. Furthermore, decreased abundance of the family *Prevotellaceae* has been associated with both end-stage renal disease [34] and Parkinson’s disease [49]. *Lawsonibacter* has been identified in fecal samples from patients with chronic obstructive pulmonary disease [52]. Our findings of higher proportional *Prevotellaceae* abundance in paired voles and higher proportional *Lawsonibacter* abundance in isolated voles are largely compatible with the general trends of our fecal analyses showing greater proportional abundances of taxa associated with better host health in paired voles and greater proportional abundances of taxa with members associated with disease in isolated voles.

### 3.5. Isolated Voles Exhibit Serum and Fecal Metabolomes Similar to Those of Animal Models for Type Two Diabetes and Colitis

Metabolite analyses have previously been used in the characterization of the fecal and serum metabolomes to create metabolomic profiles for animals subjected to different adverse stimuli or experiencing different pathological conditions. In the past, the impact of fluoxetine [53], antibiotics [54], and unpredictable stress [53,55] on the mammalian metabolome have been examined. Likewise, researchers have attempted to develop metabolomic profiles of animal models for different conditions such as fatty liver disease [56], colitis [57,58], and type two diabetes [59,60]. Our metabolite analyses displayed numerous differences between paired and isolated groups from both fecal and serum samples. Although these differences were non-significant, we deem it noteworthy that several of the pertinent metabolites have been implicated in pre-diabetes [61,62], type two diabetes [59,60,62,63], colitis [57,58], and intestinal environments that may lead to the increased virulence of intestinal pathogens [64,65]. These findings are consistent with our initial hypotheses.

Isolated voles exhibited changes in their fecal metabolomes consistent with colitis and gastrointestinal environments associated with increased bacterial pathogen virulence. Isolated voles exhibited elevated carboxylic acid metabolites (i.e., oxamic acid, tetradecanoic acid), as well as increased concentrations of several carbohydrates (i.e., arabinose, galactose, and glucose) and carbohydrate derivatives (i.e., N-acetyl-glucosamine) in their feces (Supplemental Table S1). Increased glucose intake has been found to be associated with increased susceptibility to experimentally induced colitis in mice [66]. The fecal samples from mouse models of colitis have been found to exhibit impaired carboxylic acid metabolite breakdown and increased concentrations of carbohydrate metabolites (Robinson et al., 2016). Additionally, increased fecal succinate has also been found in mice following the experimental induction of colitis (Osaka et al., 2017) and has been linked to the increased virulence of such pathogens as *Clostridium difficile* [65] and *Citrobacter rodentium* [64].

Similarly, in serum samples, notable differences in metabolites, although not statistically significant, included greater amounts of hippuric acid and lactic acid in paired voles and higher levels of palmitic acid, glyoxylic acid, and sorbitol in the isolated group (Supplemental Table S2). In humans, previous reports have identified elevated serum glyoxylate, of which glyoxylic acid is a conjugate base, to be a possible early indicator of the later development of type two diabetes [61] and elevated serum sorbitol to be a biomarker of type two diabetes [63]. An extensive characterization of metabolite biomarkers in numerous tissues and fluids in a mouse model of type two diabetes found these mice to have decreased serum levels of lactate, the conjugate base of lactic acid [59]. Additionally, lower levels of hippuric acid have been found in urine samples, although not serum samples, from individuals with impaired glucose tolerance [62]; it is unclear whether reduced hippuric acid in urine is generally accompanied by reduced hippuric acid levels in feces. Together these patterns are largely consistent with our findings of increased glyoxylic acid and sucrose and decreased lactic acid in the serum of isolated prairie voles. This suggests social isolation may alter the serum metabolome of prairie voles in a way that, at minimum, induces several features characteristic of either pre-diabetes or type two diabetes, if not lead to those actual disorders. These possibilities are consistent with previous research utilizing mouse models of diabetes that have shown chronic social isolation to contribute to disease onset [8].

### 3.6. Links between Metabolite Concentrations and Animal Behavior

Decreased time spent in the EPM open arms and immobility in the FST are indicators of stress, suggesting anxiety and depression. Positive correlations in paired animals were observed between serum metabolite concentrations of tetradecanoic acid and butanoic acid with time spent in the open arms of the EPM, as well as fecal tetradecanoic acid and immobility in the FST. Both tetradecanoic acid and butanoic acid have been studied for possible roles in mediating behaviors indicative of anxiety and depression in rodent models. Tetradecanoic acid was found to be lower in concentration under social stress, and administration of *Lactobacillus paracasei* HT6 via oral gavage has been shown to prevent its depletion [67]. Furthermore, injections of mixtures of fatty acids containing tetradecanoic acid [68] and injections of tetradecanoic acid [69] have previously been found to decrease behavioral indicators of anxiety in rats. Together, these studies suggest that tetradecanoic acid may have an anxiolytic effect in rodent models and that concentrations of this metabolite can be influenced by both social stress and the gut microbiome. However, the relationship between butanoic acid and behavioral measures of anxiety and depression appears to be less clear. The supplementation of drinking water with sodium butyrate has been shown to decrease the immobility time in the FST exhibited by socially stressed mice, as well as reduce some behaviors indicative of anxiety in such animals, but not others [70]. Conversely, when injected, sodium butyrate has been shown to increase immobility time in the FST when administered to mice multiple times within a single day, but not when administered regularly over an extended period [71]. The differences observed in previous studies may potentially, in part, be due to differences between those studies in how the metabolite was administered to animals and the behavioral assessments implemented by researchers.

The results of our study regarding the possible relationships between these metabolites and behaviors associated with anxiety and depression are consistent with some, but not all, of these previous studies. Specifically, our findings that serum tetradecanoic acid in paired voles is positively correlated with time spent in the open arms of the EPM are consistent with work demonstrating tetradecanoic acid’s anxiolytic effects [68,69], although it is unclear why fecal tetradecanoic acid in paired animals was positively correlated with immobility in the FST. Additionally, our findings that serum butanoic acid in paired animals is positively correlated with increased time in the open arms of the EPM is consistent with work demonstrating the metabolites previously reported anxiolytic effects [70]. Hence, we suggest that our correlations pertaining to the effects of tetradecanoic acid and butanoic acid support their possible anxiolytic properties, although given that these correlations were only observed for paired animals, we note that these correlations may be indicative of differences in how these metabolites impact prairie voles differently when living under stressful versus control conditions.

### 3.7. Limitations and Future Directions

The present study indicates that following four weeks of social isolation the gut microbiome as well as gut and serum metabolomes of isolated prairie voles exhibit considerable differences from those of paired voles. Several of these differences suggest poorer health in isolated conditions as they involve either lower proportional abundances of taxa with members associated with benefits to host health and higher proportional abundances of taxa containing pathogens or associated with conditions such as type two diabetes, Parkinson’s disease, and various gastrointestinal disorders. Several differences in fecal and serum metabolites from our study, although not significant, support this interpretation, especially regarding type two diabetes and to some extent colitis.

Interestingly, we note that changes in microbial communities, specifically between T_0_ and T_4_ did not always coincide with the kind of changes in metabolites one might expect, perhaps the most notable being increased *Anaerobutyricum* at T_4_ did not coincide with a significant increase in any SCFAs between T_0_ and T_4_. Given that previous research on the impact of stress on the gut microbiome and metabolome has demonstrated that stress-induced changes in the gut microbiome precede changes in the fecal and serum metabolomes [55], we believe such discrepancies to be the result of our implementation of a four-week isolation period and that a longer isolation period would have resulted in metabolite changes consistent with T_4_ microbiome changes.

Collectively, these results lay the groundwork for future investigations into whether social isolation plays a role in the onset of such conditions as type two diabetes and various gastrointestinal disorders or increase the prevalence of specific gastrointestinal pathogens.

We also note that our findings should be interpreted with the understanding that this was an exploratory study to examine the impact of social isolation on social animals such as prairie voles. There may be certain confounding factors that may have affected these findings. First, since our study subjected animals to weekly fecal collections for a four-week period, it may have imposed additional stress on all animals. Secondly, animals in the paired condition were accompanied by their same-sex sibling during fecal collections to reduce stress from social isolation. Hence, this led to contributions from both animals during fecal collections. Although previous research has shown that co-housed sibling pairs do not exhibit differences in alpha diversity and generally have fecal microbiomes that are more similar to one another than to unrelated sibling pairs, differences in the microbiomes of sibling pairs can occur [24]. Additionally, although coprophagy has not been systematically studied in wild or laboratory-housed prairie voles, it is possible that they are coprophagic. Prairie voles may engage in this behavior to allow for efficient digestive processes and to compensate for potentially unpredictable diets in natural environments, as has been reported in other microtine species [72,73,74]. If prairie voles were coprophagic, combined fecal collections from siblings in paired conditions would capture similar communities and not impact our findings here. Thirdly, given that we did not note distinct clustering between time points until T_4_, continuing the study longer may have enhanced community differences that were starting to take shape, and possibly some of the differences noted between isolated and paired voles. Likewise, continuing the study longer may have also led to greater changes in metabolite concentrations consistent with the physiology of fecal community members. In our future studies, we will subject prairie voles to longer periods of isolation with a greater separation between fecal sample collections to avoid unnecessary stress on the animals.

## 4. Conclusions

The mammalian microbiome heavily influences the broader metabolome of host organisms, with many blood metabolites resulting exclusively from gut microbiome activity [75]. Furthermore, it has been demonstrated that chronic mild stress can lead to alterations in the fecal microbiome and metabolome that in turn lead to changes in the plasma metabolome that are associated with behavioral measures of depression and anxiety [53,55]. Through our study we sought to further previous work on the effects of social isolation, a serious stressor for social mammals, on the prairie vole gut microbiome and try to determine which gut and serum metabolites may help mediate changes in the neurophysiology and behavior of isolated animals. Interestingly, social isolation was broadly associated with a lower proportional abundance of bacterial taxa associated with host health and a higher proportional abundance of taxa known to contain pathogens or be associated with disease. Furthermore, it seemed to induce metabolite biomarkers of type two diabetes in the serum of isolated voles and of colitis in the feces of these animals, suggesting social isolation in social mammals may contribute to the development of these conditions and that both the microbial and metabolite markers may be used as early indicators of these and other diseases.

## 5. Methods

### 5.1. Animals and Condition Assignment

Female prairie voles (N = 22) were used in this study due to the species’ characteristic social behaviors [10,11,12] and the fact that females of this species are induced ovulators that do not ovulate until exposed to an unfamiliar male or his urine [11]. Animals came from a breeding colony at Northern Illinois University (NIU) and were housed with a same-sex sibling after they were weaned. Because our females were housed only with a same-sex sibling after being weaned, they did not have an active estrous cycle [76].

At the start of the experiment, the voles were approximately 60 days old. At this time, they were assigned to either a paired or isolated condition; that is, they were to either continue living with their same-sex sibling or in isolation for the duration of the experiment. Eleven voles were assigned to each condition. Those assigned to the paired condition continued to be housed with their same-sex sibling. Those assigned to the isolated condition were housed individually for four weeks in a separate room from their respective siblings without visual, olfactory, or auditory information from their respective siblings. All procedures were approved by the NIU Institutional Animal Care and Use Committee and followed all guidelines set forth in the *Guide for the Care and Use for Laboratory Animals*.

### 5.2. Sample Collection

Animals were acclimated to the room one week prior to the start of the study as well as weighed at the start of the study (T_0_) and the end of the study (T_4_). Fecal samples were collected at the beginning of the study (T_0_) and four additional times, once per week for the next four weeks (T_1–4_). To minimize stress on the animals, collections were performed on the day on which the voles were moved to fresh bedding. Collection procedures entailed placing the animals into a cage cleaned with ethanol and waiting one hour, after which the animals were placed back in their normal cages and fed while droppings were collected from the collection cage, aseptically. The animals were placed back in the collection cage for the collection of additional samples on the same day. Animals in the paired condition were accompanied by their sibling during collection procedures. This resulted in contributions from both animals to fecal samples in this condition. Samples were collected in sterile WhirlPak bags, immediately homogenized, and stored in ice until they could be stored at −80 °C following the final collection for the day.

### 5.3. Behavioral Testing

The elevated plus maze (EPM) and the forced swim test (FST) were implemented using protocols previously described [30] to measure behaviors associated with anxiety and depression.

#### 5.3.1. Elevated Plus Maze (EPM)

Animals were individually placed in the center square of the EPM, which was comprised of plexiglass with two open arms and closed arms. Animals could explore the apparatus freely for five minutes. The amount of time spent by each animal in the closed arms, open arms, and center of the EPM was assessed, as well as the number of center crossings. The amount of time spent in the open arms of the EPM was used as a behavioral measure of reduced anxiety. The number of center crossings was used as a measure of general locomotor activity. Two animals from the isolated condition were excluded from analyses because they fell off the apparatus.

#### 5.3.2. Forced Swim Test

Individual animals were gently placed into a plexiglass cylinder filled with room temperature water for five minutes. The durations of different behaviors were assessed. These behaviors included:

i. Swimming: coordinated fore- and hind- limb movements without breaking the surface of the water
ii. Struggling: the movement of the forelimbs in a manner that broke the surface of the water
iii. Climbing: attempts to scratch at or climb up the side of the FST apparatus.
iv. Immobility: floating with minimal or no limb movement

Swimming, struggling, and climbing were summed to provide one index of active coping behaviors. Immobility was used as an index of a helpless, maladaptive response. These two categories (active vs. passive responses) are mutually exclusive and exhaustive categories. After testing, each animal was returned to its home cage. The tank was emptied, sanitized with a diluted bleach solution, and refilled to 18 cm between trials.

Digital video recordings were conducted during the EPM and FST which were subsequently transferred into behavioral analysis software (The Observer XT 8.0, Noldus Information Technology, Leesburg, VA, USA). Final values for these behaviors were attained by averaging the analysis of two trained and experimentally blind raters.

### 5.4. Serum and Tissue Collection

Blood collection followed the procedures previously outlined [21]. In brief, animals were anesthetized using a combination of ketamine and xylazine. Blood (approximately 1 mL) was collected from the periorbital sinus with a capillary tube within two minutes of the anesthetic injection. To obtain serum, blood was centrifuged at 4 °C at 1100 g for 15 min following clotting at room temperature. Serum aliquots were stored at −80 °C.

Immediately following blood collection, each animal was euthanized via cervical dislocation followed by decapitation. Adrenal glands, hearts, and colons were collected aseptically. Organs were transferred to sterile tubes and stored in −80 °C until further processing. Adrenal glands and hearts were later weighed.

### 5.5. Microbiome Analysis

Extractions of gut community bacterial DNA from excreted fecal samples and colon fecal samples were performed using the QIAmp Power Fecal Pro DNA Kit (QIAGEN). DNA was quantified initially by Nanodrop One^C^ 1.4.2 (Thermo Fisher, Waltham, MA, USA). QUBIT4.0 (Thermo Fisher, Waltham, MA, USA) was later performed on all samples. For quality control, PCR and electrophoresis were performed on randomly selected samples to confirm the presence of the 16S rRNA gene using Primers F (AGAGTTTGATCCTGGCTCAG) and R (CCGTCAATTCMTTTRAGTTT). Afterwards, samples were shipped for sequencing to Swift Biosciences, (Ann Arbor, MI, USA).

### 5.6. Sequencing

Community DNA from excreted and colon fecal samples were sequenced for all nine hypervariable regions of the 16S rRNA gene using the xGen™ 16S Amplicon Panel v2 (IDTDNA, Coralville, IA, USA). Normalase, a proprietary chemistry that normalizes a pool of libraries to equal molarity (either 2nM or 4nM based on workflow) where each library is equally represented, was used for normalization. The concentration of the resulting Normalase pool was checked using qPCR before loading on the sequencer. Sequencing was performed using Illumina MiSeq V2 and MiniSeq (to boost sequencing yields) with 2 × 150 bp. A total of 14,183,031 reads were obtained which were then processed using Swift Biosciences’ 16S Swift Normalase Amplicon Panels and Swift Amplicon^®^ Panels APP (SNAPP) analysis workflow (https://github.com/swiftbiosciences/16S-SNAPP-py3; accessed on 20 March 2021) which applies standard quality filtering steps, DADA2 for assembly [77], and utilizes the RDP Classifier version 2.13 [78] for taxonomic assignment.

Sequence reads were deposited to NCBI with accession number PRJNA844116.

### 5.7. Microbial Community Analyses

Count tables for samples produced through the SNAPP analysis workflow were analyzed for diversity measures and community composition. Unless otherwise stated, count tables for samples from colon and fecal communities were analyzed separately and bioinformatic analyses were carried out in Python 3.9.10 [79] using Numpy 1.22.2 [80], Pandas 1.4.1 [81], Scikit-Bio 0.5.6 [82], and Scipy 1.8.0 [83]. For each data set, the compositions of fecal (N = 108) and colon (N = 22) communities were determined at the taxonomic levels of phylum, family, and genus. Data from two voles (one at T_0_ and another at T_3_) from the isolated group could not be obtained due to insufficient sample quantities. Data subsets were created from both colon and fecal data sets to examine the compositions of communities from isolated and paired voles. Fecal data subsets were further subdivided to determine the compositions of microbial communities of isolated and paired voles at each of the five time points at which fecal samples were collected (T_0–4_). The resultant data were analyzed statistically as described in Section 5.9.

### 5.8. Metabolite Identification and Profiling

Analyses of metabolites from excreted fecal samples and serum samples were performed at the Metabolomics Lab at the Roy J. Carver Biotechnology Center at the University of Illinois at Champaign-Urbana. GC/MS quantitative analyses were performed to identify long-chain fatty acids (LCFAs) and short-chain fatty acids (SCFAs) in fecal and serum samples. GC/MS-based targeted metabolite profiling analyses were performed to identify additional metabolites in fecal and serum samples. When metabolites were inconsistently detected within a sample type, values of zero were assigned a value of one-half the smallest detected quantity for that metabolite within the relevant sample type. The resultant data were analyzed statistically as described in Section 5.9.

### 5.9. Statistical Analysis

#### 5.9.1. Analysis of Behavioral Measures

Behavioral measures of depression and anxiety exhibited by paired and isolated animals were compared with *t*-tests in Python 3.9.10 [79] using Numpy 1.22.2 [80], Pandas 1.4.1 [81], and Scipy 1.8.0 [83]. Both heart:body weight ratios and adrenal gland:body weight ratios were calculated using the body weight of animals at the time of sacrifice and the organ weight at the time of collection. Comparisons of these ratios between groups were then made using *t*-tests in Python 3.9.10 [79] using Numpy 1.22.2 [80], Pandas 1.4.1 [81], and Scipy 1.8.0 [83].

#### 5.9.2. Comparison of Microbial Communities

For microbial community analyses, proportional abundance of taxa for fecal and colon communities were compared using Mann-Whitney U tests. Differences with a *p* value ≤ 0.05 and a median proportional abundance of 0.00005 or greater in animals from at least one group were initially deemed significant. These analyses were supplemented using linear discriminant analysis effect size method (LEfSe) [84]. Additional comparisons were made between communities from colon samples and fecal samples from T_4_ using Mann- Whitney U tests and LEfSe. Alpha-diversity was assessed using Shannon, Chao1, Simpson, and Simpson E analyses. Either Mann-Whitney U tests or Friedman tests were used to compare the results of each of these measures; which test was used was based on whether the groups for a given comparison were independent. Bray Curtis dissimilarity and Jaccard distance analyses were used to assess beta-diversity. Permutational multivariate analyses (PERMANOVAs) and principal coordinate analyses (PCoA) were performed to further probe which conditions differed from one another. A longitudinal analysis comparing changes in the proportional abundance of taxa in fecal communities between time points was performed using a Friedman test. The Friedman test allowed for repeated measures, within-subjects comparison (paired: N = 11 and isolated: N = 9) at multiple time points. Individuals for which data were missing from one time point were removed from analyses. When Friedman tests yielded significant results (*p* ≤ 0.05) for comparisons between different time points, Conover tests were performed to determine which time points were significantly different from one another. To better visualize changes in microbial communities within voles between time points, heatmaps were generated using Matplotlib 3.5.2 [85] and Seaborn 0.11.2 [86].

#### 5.9.3. Comparison of Metabolites

Metabolite analyses of fecal samples from paired and isolated voles were made for samples collected at T_0_ and T_4_ using Python 3.9.10 [79] with Numpy 1.22.2 [80], Pandas 1.4.1 [81], and Scipy 1.8.0 [83]. T_0_ analyses included samples from 15 of 22 voles (paired = 8; isolated = 7). T_4_ analyses included samples from 20 of 22 voles (paired = 11; isolated = 9). Exclusions from analyses at these time points were due to insufficient quantities of feces. Normality tests and tests for equal variances were performed for each metabolite in each condition. Comparisons of metabolites from paired and isolated voles were performed using either *t*-tests on untransformed data, *t*-tests on log10 transformed data, or Mann-Whitney U tests on untransformed data. Comparisons of metabolites from serum samples taken at T_4_ (N = 22; paired = 11; isolated = 11) were also made.

The test used for each metabolite comparison at each of the two individual time points was dependent upon whether assumptions of normality and equal variances were met. If untransformed data for a particular time point met these assumptions, a *t*-test was used. If these assumptions were not met, a log10 transformation was performed for those data. If the log10 transformed data met assumptions of normality and equal variances, a *t*-test was performed on the log10 transformed data for that metabolite. If these assumptions were still not met, a Mann-Whitney U test was performed on the untransformed data for that metabolite. Mann-Whitney U tests were set to calculate the exact *p* value due to small sample sizes. Because data for many metabolites failed to meet assumptions of normality and equal variances, T_0_ values were subtracted from corresponding T_4_ values for all fecal metabolites, when available, to measure changes between T_0_ and T_4_. Resulting values were assessed for normality and equal variances, then analyzed using the same pipeline as fecal samples from single time points.

Following the above statistical analyses, the Benjamini-Hochberg method was applied to the results of each set of microbiome and metabolite comparisons implementing either Mann-Whitney U tests or Friedman tests. This was done to provide insight regarding possible false positives. A false discovery rate of 0.05 was used. The raw files for each analysis have been uploaded to FigShare at https://doi.org/10.6084/m9.figshare.20452437.v1 (accessed on 17 November 2022).

#### 5.9.4. Correlations between Behavior, Microbial Communities, and Metabolites

Correlations between anxiety- and depressive- like behaviors (i.e., time in the open arm of the EPM, time in the closed arm of the EPM, and immobility in the FST) with T_4_ fecal taxa, T_4_ colon taxa, T_4_ fecal metabolites, and serum metabolites were assessed using the Pearson correlation coefficient. Correlations were deemed meaningful if the correlation coefficient was greater than ±7.5 and the *p* value was ≤0.05.

## Figures and Tables

**Table 1:** Taxa with significant differences between time points.

Taxon	Stats		Proportional Abundances				
	Q Test	*p*-Value	Median T_0_	Median T_1_	Median T_2_	Median T_3_	Median T_4_
*Clostridiaceae 1*	21.726	<0.001	0	1.0185E − 05	4.0718E − 06	0	4.8021E − 05
*Paenibacillaceae 1*	19.84	0.001	0	0	0	0	1.4675E − 05
*Planococcaceae*	16.416	0.003	0	0	0	0	0.00018553
*Bdellovibrionaceae*	16.486	0.002	1.0146E − 05	0	0	0	0.00027892
*Ruminococcus*	14.8	0.005	0.17876649	0.12738538	0.141373	0.15814367	0.07193627
*Barnesiella*	16.364	0.003	0.0001477	0.0003441	0.00039466	0.00030383	8.3054E − 05
*Millionella*	13.88	0.008	0.00031359	0.0004766	0.00047283	0.00066886	0.00013524
*Butyricicoccus*	13.055	0.011	0	1.2102E − 05	7.9223E − 05	1.3882E − 05	0.00018001
*Vampirovibrio*	16.487	0.002	2.0047E − 05	0	0	0	0.00062135
*Mediterranea*	19.304	0.001	0	0	0	0	8.0281E − 05
*Anaerobutyricum*	27.333	<0.001	0	0	0	0	2.9015E − 05
*Phocaeicola*	15.843	0.003	0	0	0	0	1.9131E − 05

## Data Availability

Sequence reads were deposited to NCBI with accession number PRJNA844116. Files containing raw metabolite data have been uploaded to FigShare at https://doi.org/10.6084/m9.figshare.20452437.v1.

## Supplementary Materials

The following supporting information can be downloaded at: https://www.mdpi.com/article/10.3390/ijms24021677/s1.

## Abbreviations

ACTH: adreno-corticotrophin hormone; ANS: autonomic nervous system; AVP: vasopressin; CRH: corticotropin-releasing hormone; EPM: elevated plus maze; FST: forced swim test; LCFA: long chain fatty acid; LefSe: linear discriminant analysis effect size method; NIU: Northern Illinois University; OT: oxytocin; PCoA: principal coordinate analyses; PERMANOVA: permutational multivariate analyses; PNS: parasympathetic nervous system; PVN: paraventricular nucleus; SCFA: short chain fatty acid; SNAPP: Swift Normalase Amplicon Panels and Swift Amplicon® Panels APP; SNS: sympathetic nervous system; T0: time 0; T1: time 1; T2: time 2; T3: time 3; T4: time 4.
