# Supplementary material for: Microbiome and Metabolome Variation as Indicator of Social Stress in Female Prairie Voles
Source: Int J Mol Sci. 2023 Jan 14;24(2):1677. doi: 10.3390/ijms24021677 (PMC9861106; doi:10.3390/ijms24021677)
Supplement: Supplementary file 1 [file ijms-24-01677-s001.zip › ijms-2075144-supplementary.pdf]

Supplemental Table 1. Differences in Fecal Metabolite between T_0 and T_4 in paired vs isolated group

			Isolated		*Paired*	
Metabolite	**Analysis**	**Results**	**Mean**	**Std Dev**	**Mean**	**Std Dev**
Tetradecanoic acid	T-test	0.034	31.971	93.363	-69.175	72.403
Xylose	T-test	0.088	3076.129	4865.721	-1488.912	4702.852
Glucose	Mann-Whitney	0.072	1530.143	4105.073	-2350.075	5014.246
Arabinose	T-test	0.071	1167.15	1341.074	-582.569	1987.693
O-phosphoethanolamine	Mann-Whitney	0.094	5.843	15.684	-16.138	25.139
Oxamic acid	Mann-Whitney	0.072	2.693	8.938	-6.35	5.961
N-acetylglucosamine	T-test	0.096	-6.929	159.384	-168.588	185.343
Adipic acid	Mann-Whitney	0.094	-10.229	39.795	-101.675	113.563

Supplemental Table 2. Differences in Fecal Metabolites at T_4.

			Isolated		*Paired*	
Metabolites	**Analysis**	**Results**	**Mean**	**Std Dev**	**Mean**	**Std Dev**
Succinic acid	T-test	0.036	1053.656	432.8449	636.882	389.3606
Tetradecanoic acid	T-test	0.011	196.067	68.9565	109.173	66.6781
Glucose	Log10	0.097	7614.767	3822.3902	5272.345	4545.8686
Arabinose	Log10	0.088	3755.389	2600.9074	2163.568	1309.5832
Galactose	Log10	0.099	1444.178	812.4141	937.555	774.5478
Hippuric acid	Log10	0.064	418.278	572.0355	639.118	497.2158
N-Acetyl glucosamine	Mann-Whitney	0.08	236.722	101.5849	161.673	71.0349
Oxalic acid	T-test	0.091	43.411	19.4825	31.355	10.0978
O-phosphoethanolamine	T-test	0.082	36.933	10.0652	29.382	8.3091

Supplemental Table 3. Differences in Serum Metabolites at T_4 in paired vs isolated group.

			Isolated		*Paired*	
Metabolite	**Analysis**	**Results**	**Mean**	**Std Dev**	**Mean**	**Std Dev**
Hippuric acid	T-test	0.04	12.5	6.8178	19.627	8.3211
Lactic acid	Log10	0.057	10865.845	3496.8387	13662.927	3435.1915
Sorbitol	T-test	0.053	60.624	22.7011	43.145	15.6869
Glyoxylic acid	Mann-Whitney	0.088	23.618	13.1432	12.836	9.7589

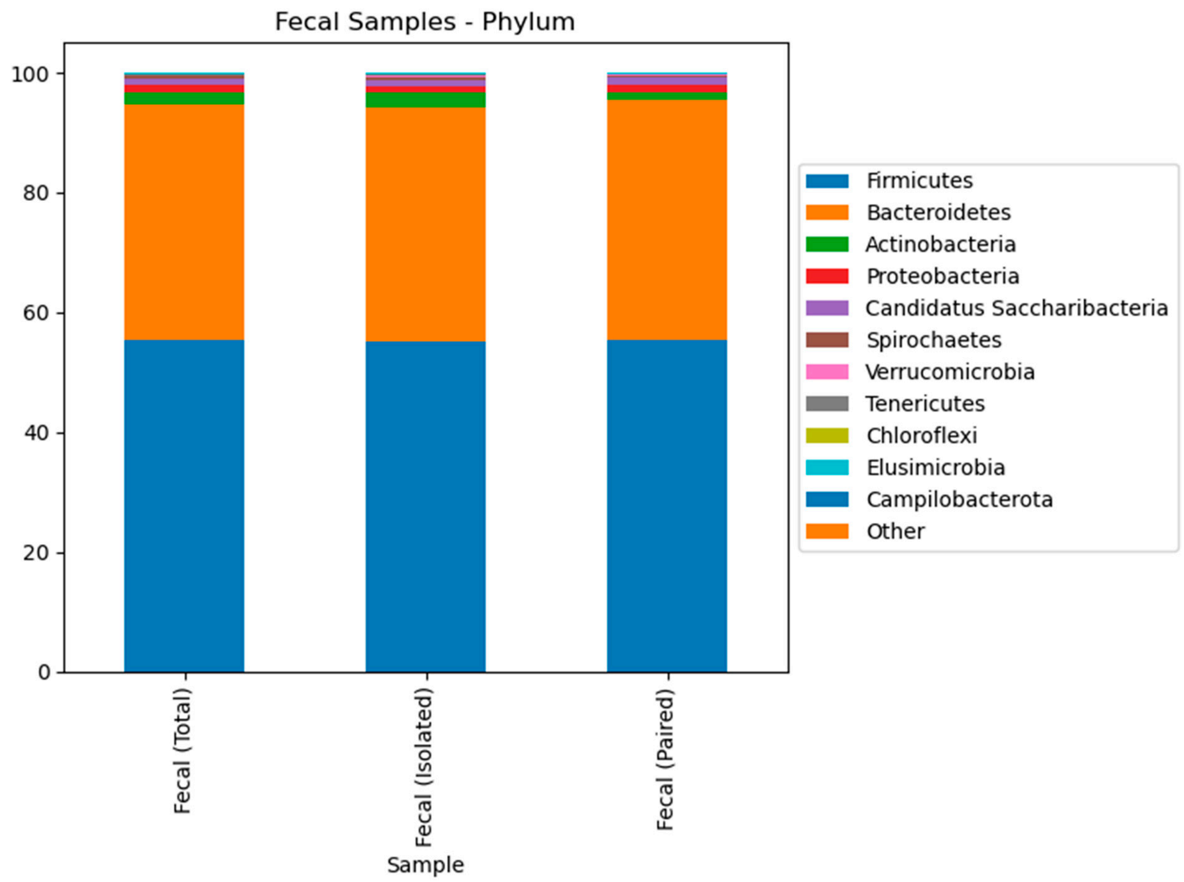

Supplemental Figure S1. Firmicutes (55.36%) and Bacteroidetes (39.44%) were the dominant phyla in pooled prairie vole fecal communities, followed by Actinobacteria (1.82%), Proteobacteria (1.24%), and Candidatus Saccharibacteria (1.1%). All other phyla comprised less than 1% of prairie vole fecal communities. Phyla contained in "Other" made up less than 0.01% of fecal communities.

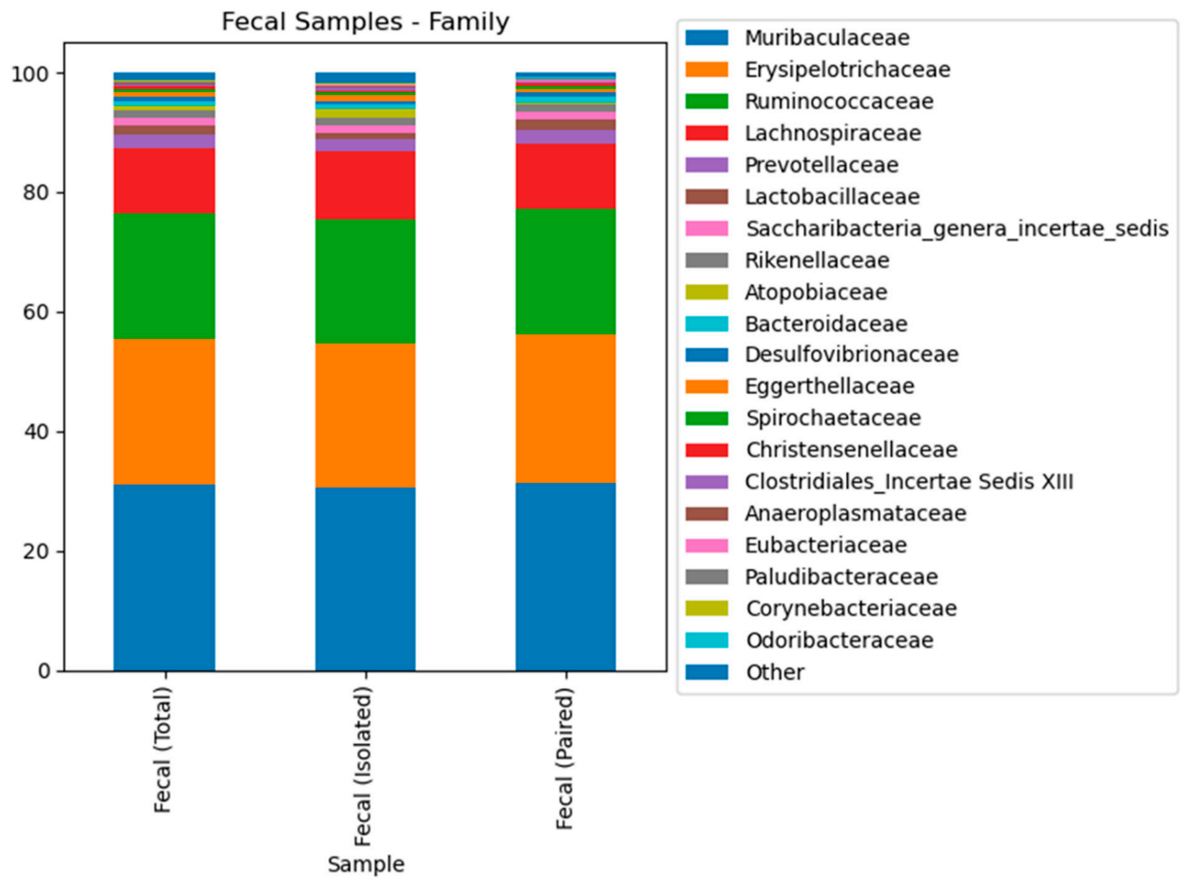

Supplemental Figure S2. Muribaculaceae (31.07%), Ersipelotrichaceae (24.44) and Ruminococcaceae (20.92%) were the dominant families in pooled prairie vole fecal communities, followed by Lachnospiraceae (11.02%), Prevotellaceae (2.14%), Lactobacillaceae (1.44%), Saccharibacteria genera incertae sedis (1.28%), and Rikenellaceae (1.24%). All other families comprised less than 1% of prairie vole fecal communities. Families contained in “Other” made up less than 0.1% of fecal communities.

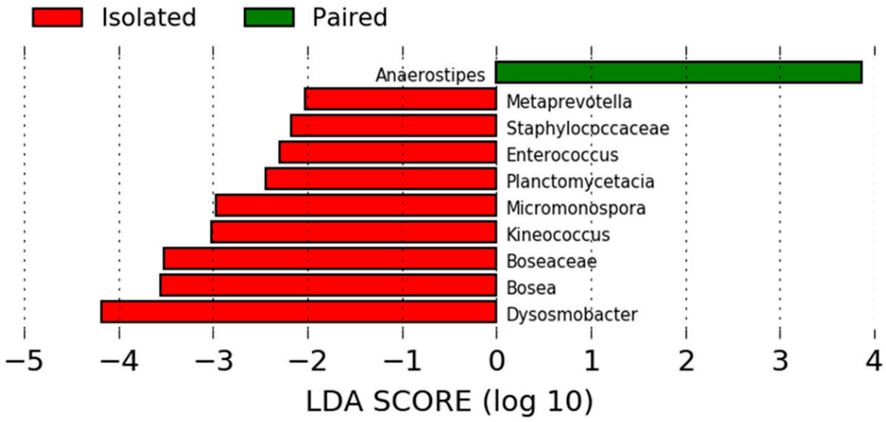

Supplemental Figure S3. LEfSe analyses revealed numerous differences in the fecal communities of paired (N = 55) and isolated prairie voles (N = 53). Paired voles had a greater proportional abundance of Anaerostipes. Isolated voles had a greater proportional abundance of nine taxa, most notably Metaprevotella, Staphylococcaceae, and Enterococcus.

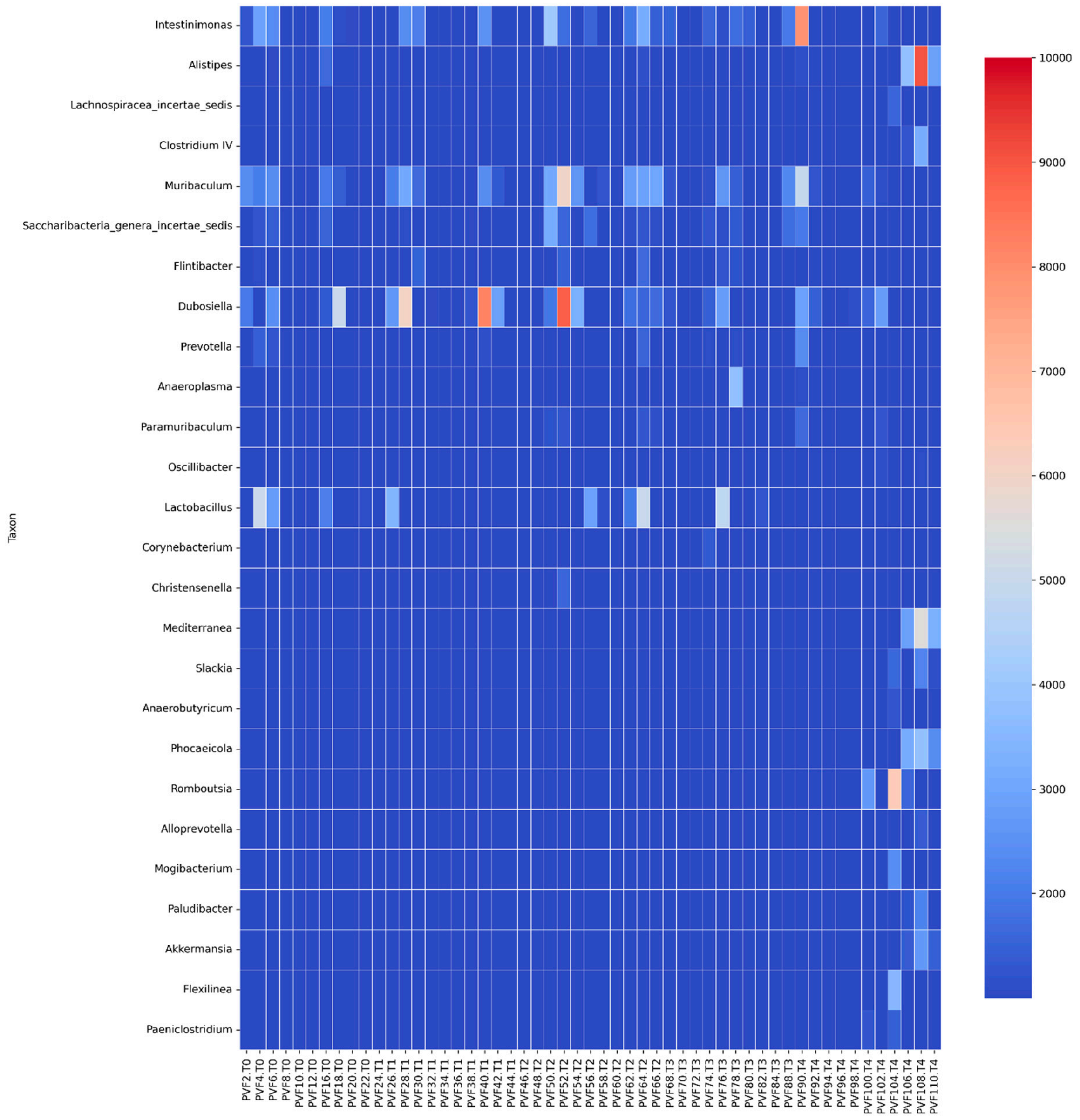

Supplemental Figure S4. Variations in taxa in isolated voles begin to emerge at T3 and T4.

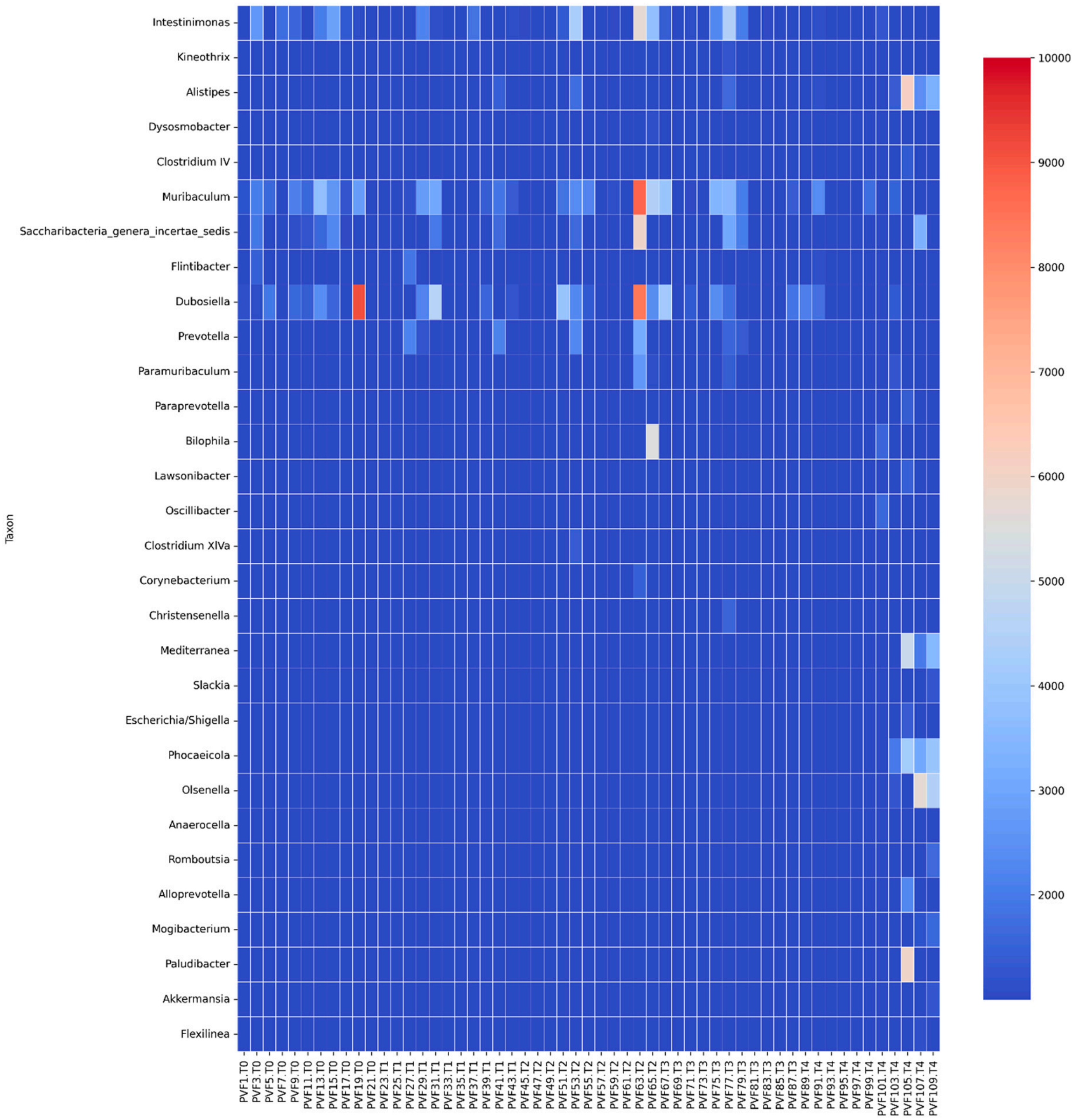

Supplemental Figure S5. Variations in taxa in paired voles begin to emerge at T3 and T4.

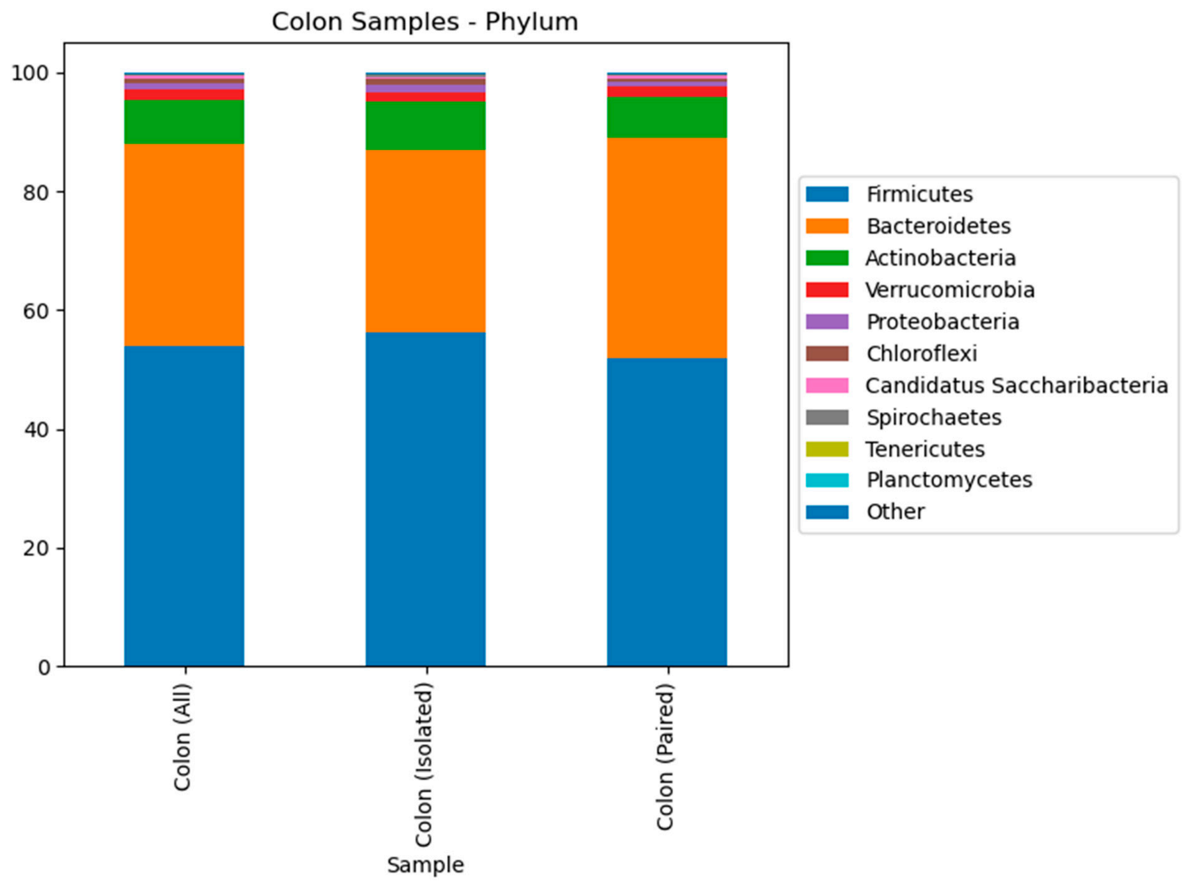

Supplemental Figure S6. Firmicutes (54.04%) and Bacteroidetes (33.895%) were the dominant phyla in pooled colon communities followed by Actinobacteria (7.496%), Verrucomicrobia (1.71%), and Proteobacteria (1.06%). All other phyla comprised less than 1% of prairie vole colon communities. Phyla contained in "Other" made up less than 0.05% of colon communities

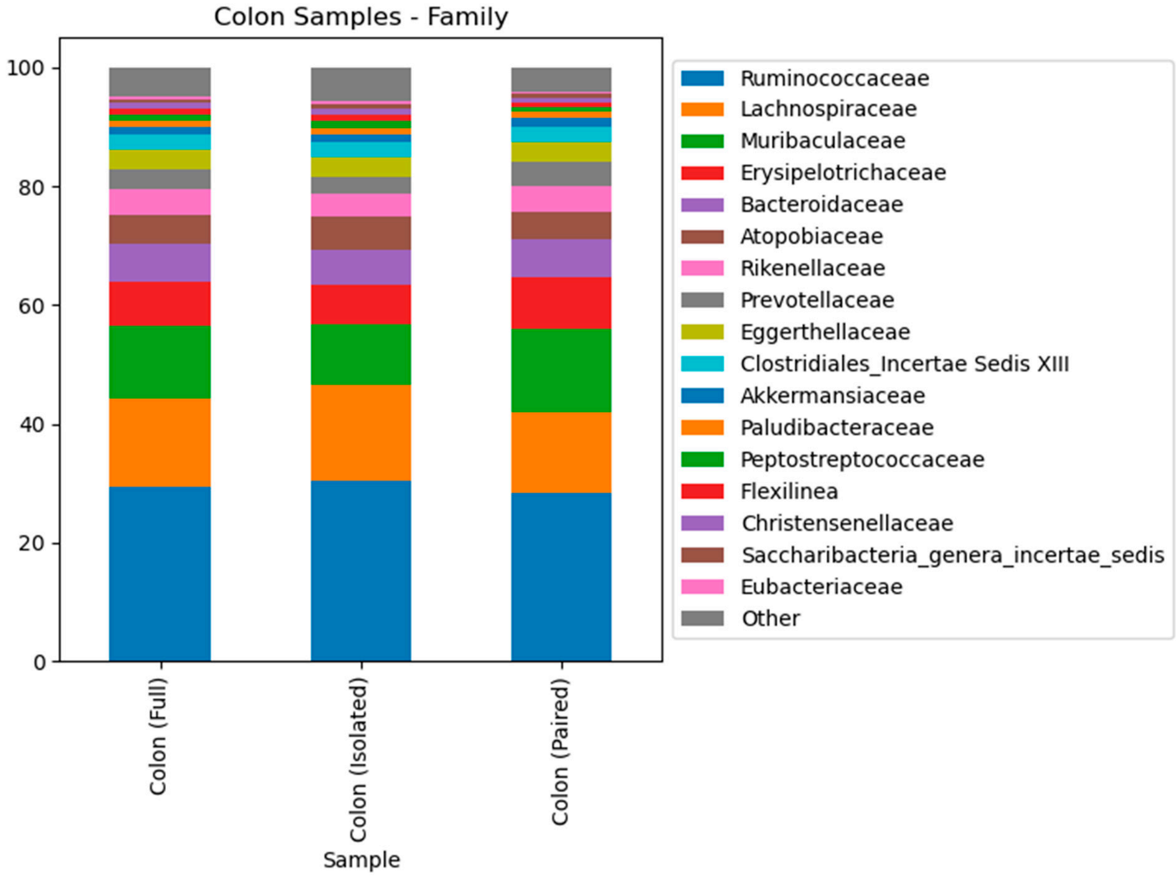

Supplemental Figure S7. Ruminococcaceae (29.34%), Lachnospiraceae (14.95%), Muribaculaceae (12.19%) were the dominant families in pooled colon fecal samples followed by Ersipelotrichaceae (7.5%), Bacteroidaceae (6.24%), Atopobiaceae (5.07%), Rikenellaceae (4.16%), Prevotellaceae (3.46%), Eggerthellaceae (3.17%), Clostridiales_Incertae Sedis XIII (2.62%) Akkermansiaceae (1.38%), Paludibacteraceae (1.05%), and Peptostreptococcaceae (1%). All other families comprised less than 1% of colon communities. Families contained in “Other” made up less than 0.1% of colon communities

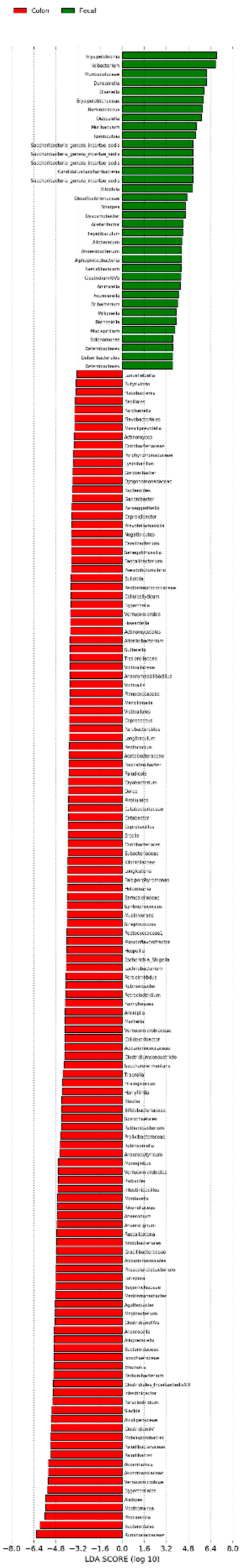

Supplemental Figure S8. LEfSe analyses demonstrated 166 differences in proportional abundances of taxa in colon (N = 22) and T4 fecal communities (N = 22) with 135 of those taxa having a greater proportional abundance in colon samples.
